# Key factor screening in mouse NASH model using single-cell sequencing combined with machine learning

**DOI:** 10.1016/j.heliyon.2024.e33597

**Published:** 2024-06-25

**Authors:** Yu-Mu Song, Jian-Yun Ge, Min Ding, Yun-Wen Zheng

**Affiliations:** a Guangdong Provincial Key Laboratory of Large Animal Models for Biomedicine, and South China Institute of Large Animal Models for Biomedicine, School of Pharmacy and Food Engineering, Wuyi University, Jiangmen, Guangdong, China; b Institute of Regenerative Medicine, and Department of Dermatology, Affiliated Hospital of Jiangsu University, Jiangsu University, Zhenjiang, Jiangsu, China; c Department of Medical and Life Sciences, Faculty of Pharmaceutical Sciences, Tokyo Univ of Science, Noda, Chiba, Japan; d Center for Stem Cell Biology and Regenerative Medicine, Institute of Medical Science, The University of Tokyo, Tokyo, Japan

**Keywords:** NASH, Macrophage, Machine learning, Single-cell RNA sequencing, Core genes

## Abstract

**Aims:**

To identify and analyze genes closely related to the progression of nonalcoholic steatohepatitis (NASH) by employing a combination of single-cell RNA sequencing and machine-learning algorithms.

**Main methods:**

Single-cell RNA sequencing (scRNA-seq) analysis was performed to find the cell population with the most significant differences between the Chow and NASH groups. This approach was used to validate the developmental trajectory of this cell population and investigate changes in cellular communication and important signaling pathways among these cells. Subsequently, high dimensional Weighted Gene Co-expression Network Analysis (hdWGCNA) was used to find the key modules in NASH. Machine learning analyses were performed to further identify core genes. Deep learning techniques were applied to elucidate the correlation between core genes and immune cells. The accuracy of this correlation was further confirmed using deep learning techniques, specifically Convolutional Neural Networks.

**Key findings:**

By comparing scRNA-seq data between the Chow and NASH groups, we have observed a notable distinction existing in the Kupffer cell population. Signaling interactions between hepatic macrophages and other cells were significantly heightened in the NASH group. Through subsequent analysis of macrophage subtypes and key modules, we identified 150 genes tightly associated with NASH. Finally, we highlighted the 16 most significant core genes using multiple iterations of machine learning. Furthermore, we pointed out the close relationship between core genes and immune cells.

**Significances:**

Using scRNA-seq analysis and machine learning, we can distinguish NASH-related genes from large genetic datasets, providing theoretical support in finding potential targets for the development of novel therapies.

## 1 Introduction

Non-alcoholic fatty liver disease (NAFLD) is a major contributor of the rising incidence of chronic liver disease globally, with about 25 % of the population worldwide suffering from the condition [1,2]. This disease is associated with the accumulation of abnormal fat in the liver, even in the absence of alcohol consumption. NAFLD is a broad term for a variety of pathologic alterations to the liver, including simple fat accumulation, ballooning degeneration, inflammation, fibrosis, and steatohepatitis, which is well-known as nonalcoholic steatohepatitis (NASH) [3]. In addition, patients with NASH suffer from varying degrees of hepatocellular inflammation and hepatocellular necrosis [4] and may further develop into cirrhosis or even liver cancer [5,6]. The pathogenesis of NASH is complex, being associated not only with fat accumulation but also closely related to inflammatory response. Despite significant advancements in understanding the pathophysiology of NASH, no particular drug has been developed to effectively treat or cure NASH [7]. In recent years, booming researches have been focused on dissecting the involvement of hepatic macrophages in the progression of NASH, hinting at the potential of hepatic macrophages as a promising therapeutic target for alleviating NASH [8].

In recent years, the application of scRNA-seq and machine learning has attracted widespread attention in the field of biomedicine. scRNA-seq enables the analysis of gene expression at the individual cell level, gaining a new perspective on the identification of key genes and signaling pathways. This novel approach reveals the heterogeneity of cells within tissues, offering a fresh perspective for understanding the molecular mechanisms underlying the pathogenesis and progression of diseases [9,10]. It is anticipated that machine learning algorithms will offer novel approaches for the early detection and treatment of NASH because of their special ability to identify complicated data patterns, such as disease-related gene expression profiles, and to make predictions from massive amounts of data [11]. In this research, we innovatively employed an extensive array of transcriptomic data, in conjunction with machine learning and deep learning methodologies, to analyze mouse models of NASH. This study is of great significance for the development of novel therapeutic strategies for NAFLD and NASH.

## 2 Material and methods

### 2.1 Data sources and pre-processing

The GEO database (https://www.ncbi.nlm.nih.gov/geo/) is an internationally recognized public repository of rich high-throughput sequencing data and has become an important platform for collecting RNA sequencing data [12]. In our study, we selected GSE129516 as the scRNA-seq data source for initial analysis from the GEO database. We utilized a total of 6 samples in our analyses, including 3 normal samples (Chow) and 3 samples from NASH conditions. It should be noted that all samples were derived from liver of C57/Bl6 mice, a commonly used strain for studying metabolic liver diseases due to their susceptibility to diet-induced steatohepatitis. scRNA-seq database analyses were done based on the R environment (v.4.2.2) and using Seurat (v4.3.0) [13]. Preset conditions required that the number of genes detected per cell ranged from 10 to 5000, while the proportion of mitochondrial genes was set to be less than 10 %. Subsequently, the data were standardized and normalized, and the top 2000 genes with highly variable characteristics were screened by the analysis of variance (ANOVA). These scRNA-seq data were subjected to principal component analysis (PCA) and batch effects were removed by the Harmony method [14]. In addition, a large amount of Bulk RNA-Seq data including GSE225616 and GSE217492 and et al. were found, which will be used for validation of deep learning and machine learning models. Meanwhile, the SVA package was utilized to remove the batch effect, thus generating more accurate Bulk RNA-Seq data [15] (Table S1).

### 2.2 Cell annotation and identification of key cells

The UMAP technique and the first 15 principal components (PCs) were chosen for an overall dimensionality reduction study of the preprocessed data. To guarantee the accuracy of cell annotation, we utilized the FindAllMarkers function to identify differentially expressed genes (DEGs) in each cluster. MouseRNAseqData data from the SingleR package was also used to aid in the annotation of the downscaling results [16]. Finally, in conjunction with the CellMarker database and previous studies, 11 types of cells were identified [17]. By counting the percentage of different cells in the control (Chow) and NASH groups, we identified key cell populations and performed scale mapping using the ggplot2 package.

### 2.3 Inference and analysis of intercellular communication

To gain a deeper understanding of the complex mechanisms of intercellular communication, we employed the CellChat software package as the main tool, which is specifically designed to infer and reveal intercellular communication networks [18,19]. By utilizing ligand-receptor pairs in CellChatDB, we performed a detailed analysis of the interactions between NASH and cellular populations of healthy individuals and compared NASH-related pathways.

### 2.4 Screening of candidate genes

We identified cell populations with significant disparities in NASH, followed by conducting a pseudotime analysis using the Monocle2 package. The investigation of the temporal dynamics and developmental trajectories of these unique cell populations in the setting of NASH was made easier by this methodological approach [20,21]. Firstly, cell subtype analysis was performed to identify subpopulations of cells that differed significantly between the control and NASH groups. Next, co-expression network analysis was performed on selected key cells using the hdWGCNA package. This tool is capable of inferring gene networks, identifying gene modules, and visualizing data [22]. We chose a soft power value of 4 and used the ConstructNetwork function in hdWGCNA to identify the co-expression information of the genes. Then, we determined the correlation between Chow and NASH groups to find the key modules related to NASH. 150 candidate genes affecting NASH were found by combining with subpopulations of cells with large differences.

### 2.5 Machine learning identifies core genes

Univariate logistic regression is a powerful tool for binary outcome analyses, ideally suited for scenarios where the outcome variable is dichotomous (e.g., presence/absence of disease, event/no event). This method not only provides insights into the strength and direction of the impact of each predictor on the outcome but also calculates the probabilities of these outcomes. This is crucial for understanding the importance and mechanisms by which variables influence the outcome. Bulk RNA-Seq data were employed as both training and validation sets in our analysis. Beginning with a pool of 150 candidate genes, univariate logistic regression analysis was performed on each gene using the glm function in R, calculating the odds ratio and its 95 % confidence intervals. Genes with a p-value less than 0.05 were considered to have a significant impact, reducing the number of candidate genes from 150 to 77. Following the univariate logistic regression screening, Least Absolute Shrinkage and Selection Operator (LASSO) regression analysis was conducted on the data within a binomial distribution framework using the glmnet package. A 10-fold cross-validation method was utilized to determine the optimal regularization parameter λ by minimizing cross-validation error. This approach culminated in the identification of 16 core genes. LASSO regression is a technique that applies L1 penalization to reduce regression coefficients to zero, effectively addressing multicollinearity and overfitting in high-dimensional data. This method not only simplifies the model but also enhances its predictive accuracy and interpretability. The mlr3verse package was applied to analyze each gene using a range of machine learning algorithms, including Support Vector Machine (SVM), Iterative Dichotomiser 3 (ID3), Weighted k-Nearest Neighbors (KKNN), Logistic Regression, Random Forest, Decision Tree, and Naive Bayes. Predictive analysis was performed with a 5-fold cross-validation, repeated 10 times, where SVMs demonstrated the most effective prediction results. The predictive efficacy of these 16 core genes was also validated.

### 2.6 Validation of the correlation between core genes and immune cells

The ssGAVA scores of core genes within Bulk RNA-Seq data were validated using the gsva function of the GSVA package. Their differential expression between Chow and NASH groups was assessed employing the Wilcoxon test [23]. The Wilcoxon test is an appropriate choice for our dataset, given the small sample size and the potential non-normal distribution of the data. The immune infiltration of various cell types was evaluated utilizing the deconvo_mcpcounter function in the IOBR package [24]. Subsequently, the correlation between the core genes and the degree of immune cell infiltration was determined using Spearman's rank correlation test, implemented through the corr.test function in the psych package. The Spearman test does not make specific assumptions about the distribution of data, making it more suitable for bioinformatics data, particularly when the data do not follow a normal distribution. Corrections for P-values were made using the FDR approach to address multiple testing considerations. We have also detailed the importance of using the FDR method to adjust p-values, which helps to control the risk of false positives in multiple comparisons.

### 2.7 Deep learning validation of immune cell correlations

To construct a feature matrix for deep learning training, we calculated the ratio of the proportion of immune cell types to gene expression in each sample. This step was designed to develop a model capable of transforming complex gene expression patterns into a format amenable to deep learning algorithms. Subsequently, a Convolutional Neural Network (CNN) model was constructed using the keras package in R. The CNN model incorporated a convolutional layer with 32 filters, followed by another convolutional layer with 16 filters. The model underwent optimization over 250 training iterations, with accuracy serving as the primary evaluation metric. Model performance during training was assessed using learning curves and Receiver Operating Characteristic (ROC) curves.

## 3 Results

### 3.1 Single cell transcriptome assay

**Fig. 1:**
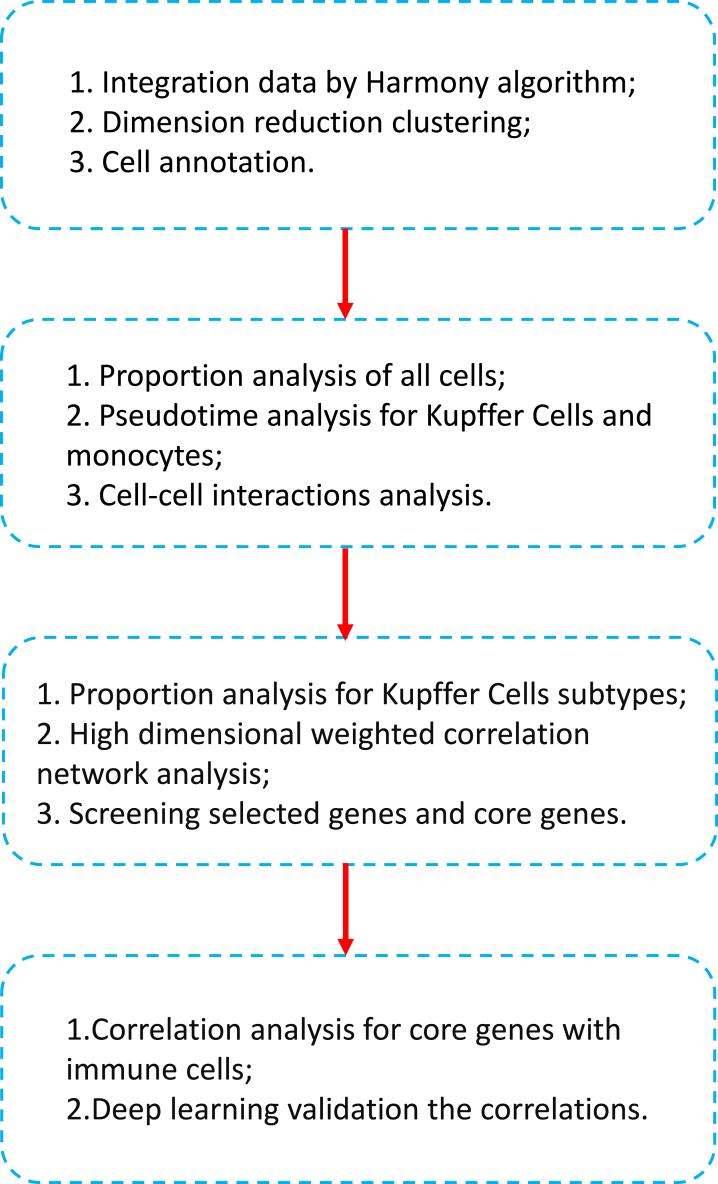
The flowchart of key factor screening in mouse NASH model.

The study design workflow was illustrated in Fig. 1, and the acquired single-cell RNA sequencing data were subjected to standard quality control measures (Supplementary Fig. S1A). After pre-processing, we applied a harmony algorithm integration to effectively merge and mitigate batch effects. This integration process, following the harmony algorithm, involved 6 samples (Supplementary Fig. S1B). The first 15 principal components (PCs) and batch processing based on Harmony were used for downstream analysis (Supplementary Fig. S1C). Following the conventional Seurat workflow, we successfully identified 13 distinct clusters, which were subsequently embedded in a UMAP (Supplementary Fig. S1D). Finally, the consolidated dataset was used for further analysis.

**Fig. 2:**
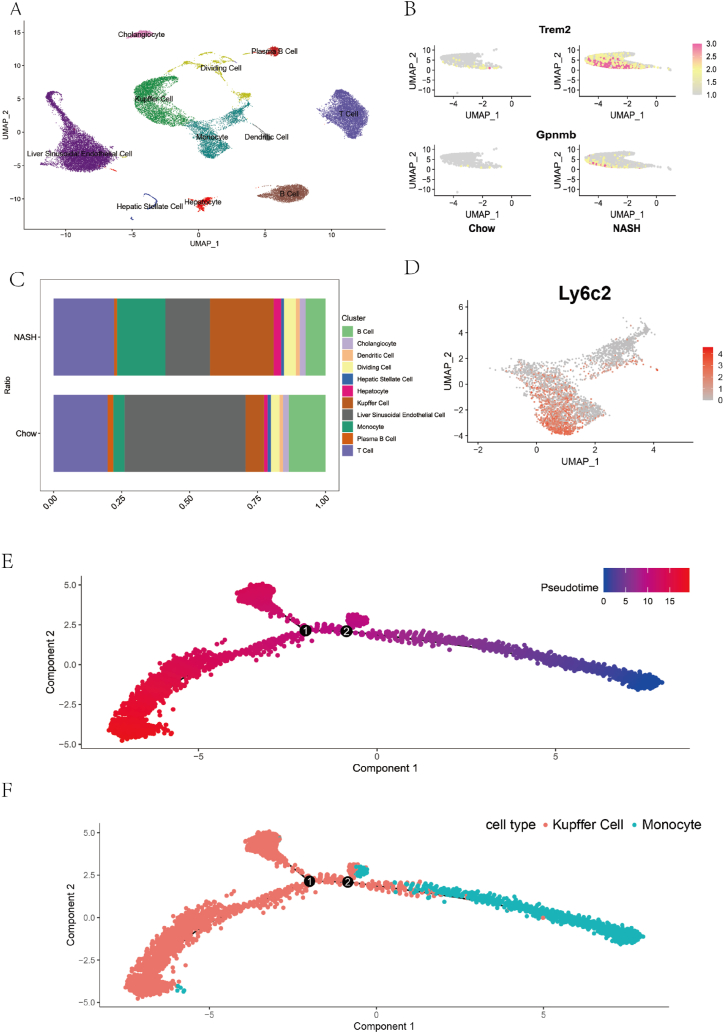
Comparison of single-cell sequencing data between chow and NASH mouse livers. (A) UMAP plot displays various cell types and their distribution; (B) Expression of *Trem2* and *Gpnmb* genes in NASH and Chow groups; (C) Proportion of cell distribution in NASH and Chow groups; (D) Expression of the *Ly6c2* gene in monocytes. (E, F) Pseudotime analysis of monocytes and Kupffer cells, with Kupffer cells being at a later developmental stage and monocytes at an earlier stage.

Mouse single cell sequencing data from the GEO database (GSE129516) were pre-processed to obtain 32,322 cells, of which the control group (Chow) had 17,482 cells and the experimental group (NASH) had 14,840 cells. We used UMAP for data downscaling, clustering and visualization. Form the 13 clusters identified, 32322 cells were annotated into 11 cell types including liver sinusoidal endothelial cells (LSECs), hepatocytes, monocytes, Kupffer cells, hepatic stellate cells, B cells, T cells, plasma cells, cholangiocytes, Dividing Cells and dendritic cells by analyzing the marker genes of each cluster (Fig. 2A). As shown in the cell ratio graph (Fig. 2C), compared with the chow group, the NASH group showed a significant decrease in the proportion of LSECs, which may be related to their transformation into a more 'capillarised' morphology. This transformation, characterized by the production of inflammatory and pro-fibrotic mediators and LSEC-mediated angiogenesis, was supposed to have a significant impact on disease progression [25]. In contrast, the proportions of Kupffer cells and monocytes were significantly increased in the NASH group. Liver macrophages primarily comprise two types: Kupffer cells and monocyte-derived macrophages. Kupffer cells, originating during embryonic development, maintain their population through self-renewal [26,27]. In the context of NASH, monocytes are recruited and transformed into macrophages to replenish the Kupffer cell pool. A characteristic feature of monocyte-derived macrophages is the expression of *Trem2* and *Gpnmb* (Fig. 2B) [28]. It has been reported that in NASH, a subset of Kupffer cells originates from *Ly6C*+ monocytes, and these monocyte-derived Kupffer cells manifest a more pro-inflammatory state in contrast to embryonic-derived Kupffer cells [26]. Accordingly, in our analysis, the expression of *Ly6c2*, a specific isoform of the *Ly6C* family, was detected in monocytes in the NASH group (Fig. 2D). Furthermore, through the implementation of an advanced pseudotime analysis proposed in our study, we discovered that Kupffer cells are situated at the terminal stage of the developmental process. This insight clarifies their specific developmental position along the differentiation trajectories(Fig. 2E–F)and also suggest that monocytes may participate in the inflammatory response through migrating to the liver and transforming into macrophages [29]. Taken together, these findings suggests that Kupffer cells play a critical role in the aetiology of NASH [30].

### 3.2 Enhanced cellular interactions of Kupffer cells

**Fig. 3:**
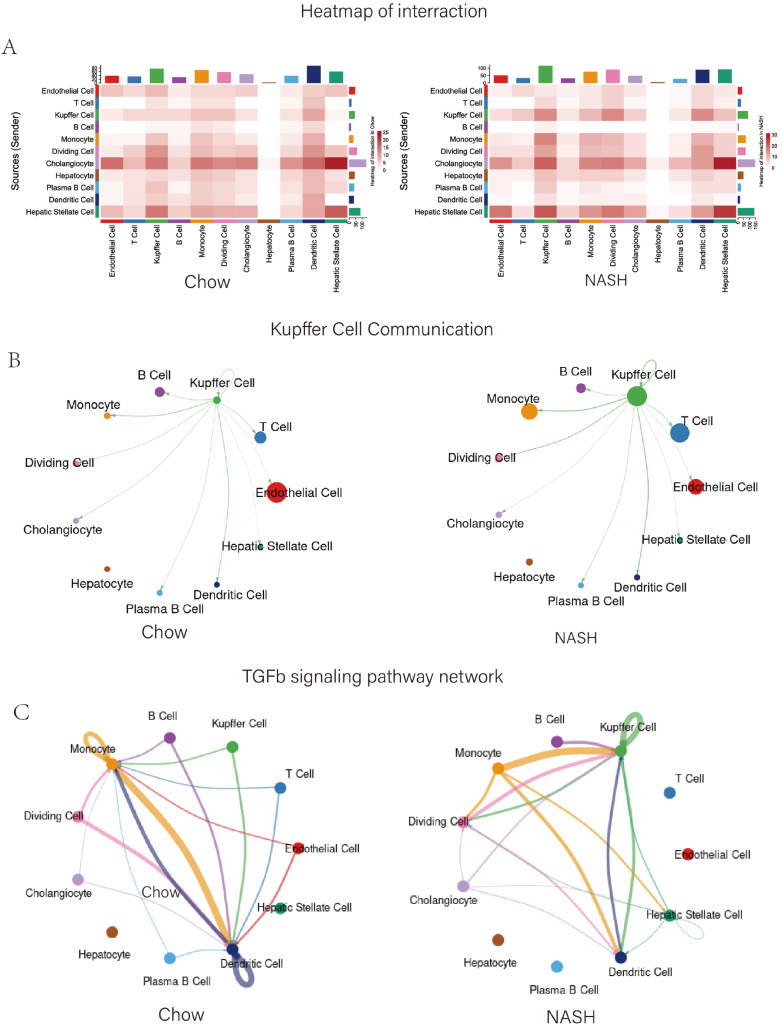
Analysis of intercellular interactions shows increased macrophage signaling in NASH. (A) Heatmap showing an overview of intercellular interactions in the Chow and NASH groups; (B) Analysis of Kupffer cell interactions with other cells in NASH and Chow groups; (C) Differences in TGF-β signaling between NASH and Chow groups are shown.

We utilized the CellChat software package for ligand-receptor pair interaction analysis and gain insight into the distinctions between the NASH and Chow livers. We analyzed datasets form each group and discovered noteworthy alterations in cellular interactions within the NASH liver, particularly markedly increased signaling between Kupffer cells and other immune cells (Fig. 3A). Special attention was given to the interactions between Kupffer cells and other cells in Chow and NASH livers, revealing that macrophages in NASH exhibited a higher propensity for communication with other cells, especially monocytes and T cells (Fig. 3B). By comparing TGF-β signaling in NASH and Chow groups (Fig. 3C), we found that Kupffer cells more actively communicated with hepatic stellate cells through TGF-β signaling, which was reported to activate the expression of genes involved in liver fibrosis [31]. This pathway was also reported to be involved in chronic liver injury during the progression of NASH, and ultimately lead to hepatocellular carcinoma (HCC) [32]. Through cellular communication analysis, we not only identified multiple communication patterns specific to NASH, but also compared these patterns with those in normal liver, thereby revealing crucial signaling changes during NASH development. These findings implied that Kupffer cells and their inter-cellular signaling may play a critical role in the progression of NASH. Consequently, we decided to select Kupffer cells as the candidate population for further analysis.

### 3.3 Selection of candidate genes

**Fig. 4:**
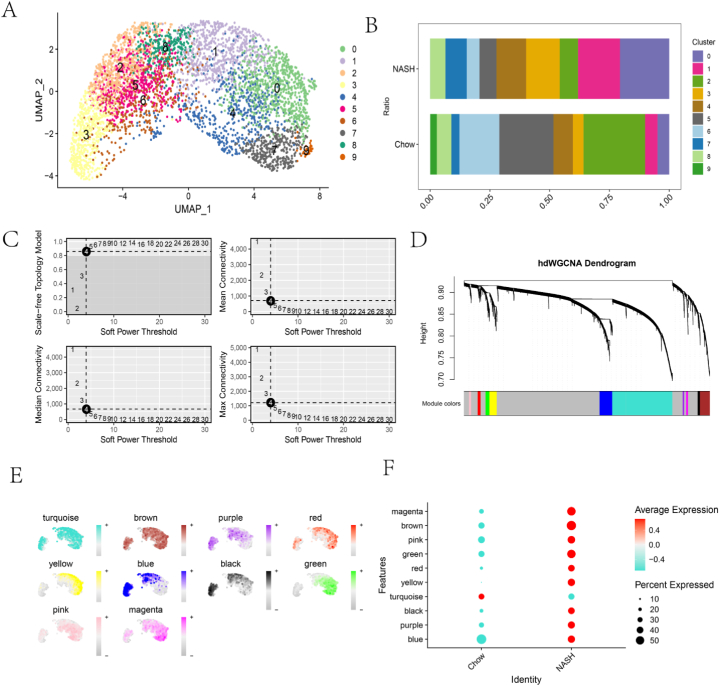
Identification of candidate genes in Kupffer cells. (A) Subpopulation analysis of Kupffer cells; (B) Proportion of cells in NASH and Chow for each subpopulation of Kupffer cells; (C) Value of Soft Power in hdWGCNA selected to be 4; (D) Construction of co-expression network; (E) 10 modules identified by hdWGCNA analysis; (F) Correlation of modules within Chow and NASH groups.

Given the above results, Kupffer cells were selected for re-analysis by UMAP. The analysis identified a total of 10 distinct clusters (Fig. 4A). The scaling diagram of the subpopulations (Fig. 4B) revealed considerable variation of the Kupffer cell subpopulations between Chow and NASH groups. To further elucidate these differences, clusters 0, 1, 3 and 7, which showed significant differences, were selected for high-dimensional gene co-expression network (hdWGCNA) analysis. A soft power value of 4 was selected to construct the co-expression network (Fig. 4C). Based on the scale-free network structure, the final analysis resulted in 10 modules, with the exception of the grey module. (Fig. 4D). These modules were found to vary in terms of the co-expression pattern engaged in different cell clusters (Fig. 4E). Subsequent analysis revealed notable discrepancy s in the expression of different modules (Fig. 4F). Considering variations in the proportions of Kupffer cell subpopulations, 6 modules - 'brown', 'yellow', 'magenta', 'green', 'black' and 'red' - were selected. The top 25 genes from each module were selected as candidate genes for further analysis.

### 3.4 Core genes identification by machine learning

**Fig. 5:**
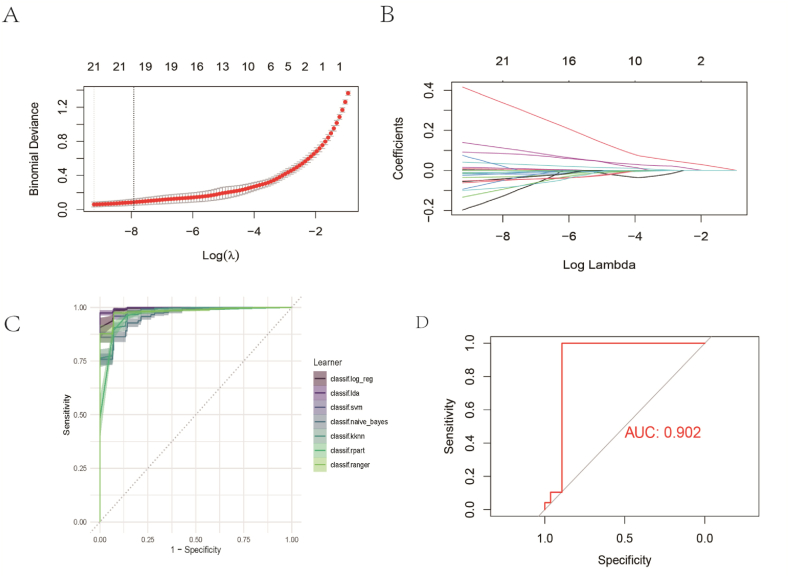
Establishing core genes through machine learning. (A) Univariate Logistic Regression for Screening Candidate Genes; (B) LASSO logistic regression algorithm optimizes core genes, showing the trend of coefficient changes for different genes with the regularization parameter λ; (C) Comparison of sensitivity and specificity among different machine learning algorithms. (D) ROC curve for the predictive results of SVM on core genes.

We acquired an extensive collection of bulk RNA-Seq data from the GEO database, serving as both training and test sets in our machine learning analysis. One-way logistic regression analysis was employed to assess the influence of individual genes on the classification of Chow and NASH groups. Logistic regression analysis was performed for each gene, and a total of 77 genes were obtained from the candidate genes (p < 0.05). (Table S3). After removing the ribosomal genes, LASSO regression analysis was conducted to selected 16 significant core genes from the statistically selected genes, including *Gpnmb* and *Lgals1* and et al. (Table S4) (Fig. 5A–B). In some studies, the overexpression of *Gpnmb* improved hepatic steatosis and fibrosis in a diet-induced obesity model, and that *Gpnmb* interacts with calnexin in liver macrophages, leading to a reduction in oxidative stress [33,34]. The results of the other study showed that when mice lacked the *Lgals1* gene, they showed a degree of resistance to high fat diet-induced obesity. The *Lgals1* gene plays an important role in adipocyte differentiation and the development of obesity, particularly through its interaction with and influence on the activity of PPARγ [35]. This conclusion demonstrates the reliability of our core gene selection. To enhance the precision of our core gene prediction, we compared multiple machine learning approaches for prediction. The comprehensive evaluation, considering accuracy, sensitivity, specificity, and error rate, revealed that the support vector machine (SVM) yielded the most favorable prediction results. This outcome may be attributed to SVM's robust capability in handling high-dimensional sparse datasets [36]. (Fig. 5C). These results provided valuable experience in machine learning model selection and validated the accuracy of our selected 16 core genes by SVM (Fig. 5D), indicating the reliability of machine learning predictions. Overall, we have identified core genes involved in NASH, which may serve as potential biomarkers or therapeutic targets for NASH treatment.

### 3.5 Validation of correlations between core genes and immune cells

**Fig. 6:**
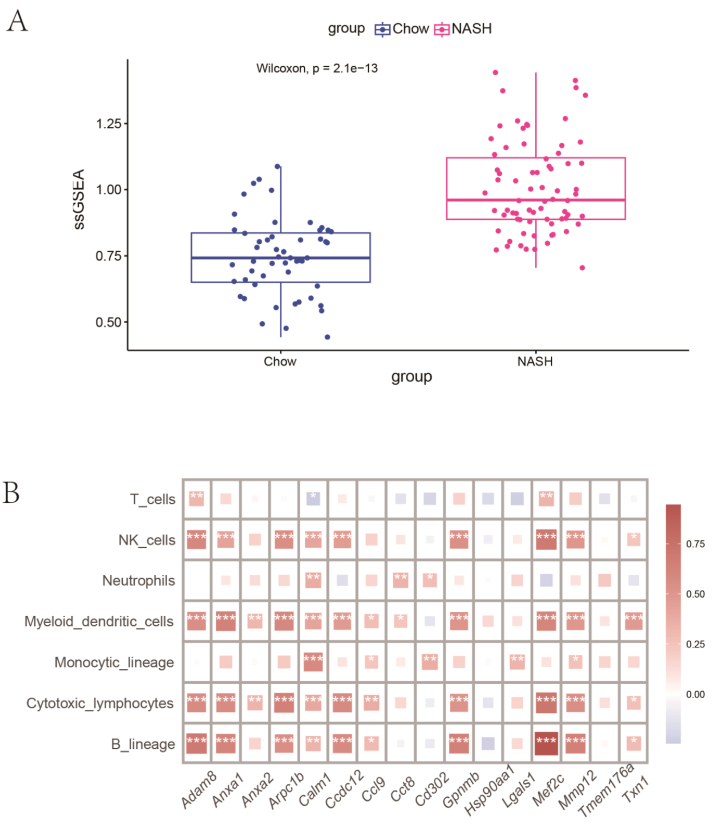
Comprehensive Validation of Core Gene Correlations with Diverse Immune Cell Subtypes. (A) Displayed are the results of the single-sample gene set enrichment analysis (ssGSEA) scores for different groups; (B) A heatmap of the correlation between selected core genes and various immune cell subgroups, such as T cells, natural killer (NK) cells, neutrophils, etc. (*P < 0.05, **P < 0.01, and ***P < 0.001).

Gene set variation analysis (GSVA) of the core genes was conducted at the bulk RNA-Seq level. The results indicated a significantly higher gene set enrichment score in the NASH liver compared to the Chow liver, suggesting that these core genes may play a crucial role in NASH development. (Fig. 6A). To understand the association between the core genes and various immune cells, we calculated the Spearman rank correlation between the expression of the core genes and immune cells and corrected it with multiple testing (Fig. 6B). The results indicated that almost all core genes were significantly associated with different immune cells. Specifically, *Adam8* and *Mmp12* were found to be highly correlated with a variety of immune cells. Additionally, *Mef2c* exhibited a notable association with B lineage cells, cytotoxic lymphocytes and myeloid dendritic cells. This finding aligns with previous reports highlighting the critical role of *Mef2c* in orchestrating the pro-inflammatory phenotype of macrophages, enabling them to effectively combat infection and generate inflammatory responses [37]. The above results indicated a highly significant correlation between core genes and immune cells in NASH.

### 3.6 Deep learning validation of immune correlations

**Fig. 7:**
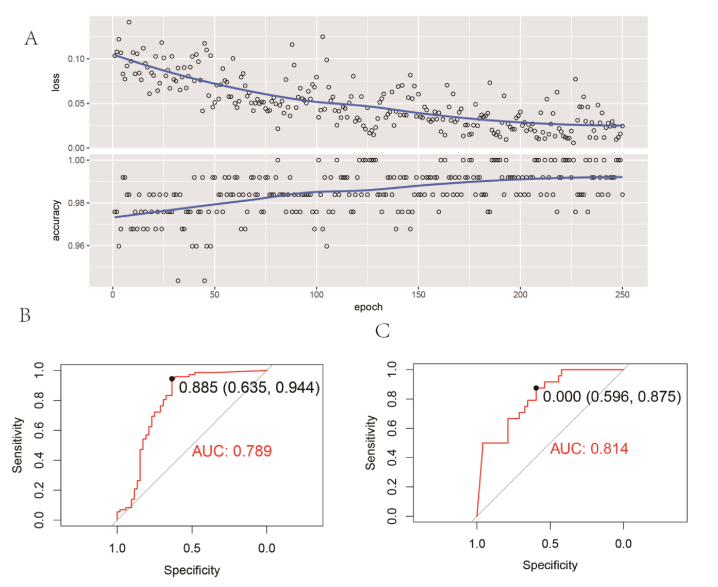
Deep Learning Validation of Immune Relevance (A) The training trajectory of CNN illustrating the dynamic changes in loss and accuracy over successive training epochs. (B, C) Displays the predictive performance of the model on the training and test sets, evaluated by the receiver operating characteristic (ROC) curve.

We further employed a convolutional neural network (CNN) to analyze gene expression data related to NASH. The graph shows that as the number of training epochs increases, the loss value gradually decreases and the accuracy increases, indicating the model's progress in learning. Following multiple training iterations, we found that a training cycle of 250 iterations yielded optimal results (Fig. 7A). To independently evaluate the predictive performance of the models on both the training and test sets, the fitting process and the receiver operating characteristic (ROC) curves of the models were illustrated (Fig. 7B and C). The area under the ROC curve (AUC) provides an overall measure of model performance, with the curve representing sensitivity and specificity at different classification thresholds. The AUC value for training set is 0.789, and for test set is 0.814, both indicating that the model has a high predictive efficacy in distinguishing immune relevance. These results suggested that deep learning approaches could efficiently decipher NASH-related patterns from gene expression data, holding potential for application in NASH diagnosis.

## 4 Discussion

NASH is a disease characterized by inflammation and liver damage that can eventually progress to cirrhosis and even liver cancer [38], but the pathogenesis of NASH is not fully understood and requires in-depth research. In this study, we employed scRNA-seq technology combined with machine learning to extensively investigate the gene expression changes in NASH, aiming to offer new insights into the pathogenesis of NASH.

In this research, we have innovatively employed scRNA-seq coupled with machine learning to perform a comprehensive analysis of the molecular mechanisms of pathogenesis in mouse NASH for the first time. We found that the proportions of Kupffer cells and monocytes were significantly increased in NASH mice, suggesting that these two immune cells play a key role in the pathogenesis of NASH. This finding was consistent with the report from Frank Tacke et al., who found that macrophages play a key role in the inflammatory response in the pathogenesis of NASH [39]. In addition, our proposed pseudotime analysis further unveiled the dynamic development of monocytes and Kupffer cells throughout the disease course, enhancing our understanding of their role in disease progression. Utilizing CellChat analysis, it revealed a notably enhanced interaction between Kupffer cells and other cellular types in the NASH group, especially monocytes. Moreover, the TGF-beta signaling pathway was found to be markedly activated in the NASH group, indicating that Kupffer cells may play a significant role in the progression of NASH. Moreover, using high-dimensional gene co-expression network analysis and machine learning methods, we identified a set of core genes associated with NASH pathogenesis, which may play key roles in cellular inflammatory responses, immune regulation and other processes, and are considered critical factors in the pathogenesis of NASH [40]. Of note, we uncovered a robust correlation between these core genes and various immune cells, underscoring their potential involvement in the activation and regulation of immune cells in the context of NASH. Building on these insights, we further investigated the presence and diagnostic relevance of proteins encoded by these core genes in peripheral blood. In our study, we confirmed the presence of several proteins associated with core genes, such as ADAM8, ANAX1, and ANXA2, in peripheral blood samples. These findings not only support the potential of peripheral blood as a medium for reflecting hepatic conditions but also highlight the utility of these proteins as non-invasive diagnostic tools. For proteins like CCDC12 and MEF2C, which were not detected in peripheral blood, further research is needed to explore their potential therapeutic value.

This study attempts to combine single-cell RNA sequencing with machine learning and innovatively uses extensive Bulk RNA-Seq data as both training and test sets to identify core genes associated with NASH in mice. The application of one-way logistic regression analysis to assess the impact of individual genes has proven to be an effective strategy, allowing rapid screening of candidate genes. The integration of LASSO regression further fine-tuned the identification of selected genes, thus simplifying the model and improving its explanatory power. However, this approach could also lead to an over-reliance on the dataset used for model training, leading to a reduction in the precision of core gene selection and the generalizability of the model. We innovatively implemented deep learning models with convolutional neural networks, which are known for their remarkable ability to handle complex and high-dimensional datasets. These advanced techniques allowed us to efficiently extract relevant features from extensive gene expression data. However, a major challenge of deep learning models is their interpretability: the complexity of these models made it difficult to directly discern the biological significance of gene expression data from the model outputs, somewhat limiting our ability to deeply understand the pathomechanisms of NASH.

Although we used machine learning techniques to identify key genes in NASH and found some consistent results with previous mouse NASH studies, this demonstrates the feasibility of this machine learning approach to some extent. However, there are still limitations to our method that need to be further validated in the future. Firstly, the findings from our mouse models may not fully represent human NASH and the core genes identified may not have the same relevance in human disease. The accuracy of our selected mouse NASH core genes is uncertain and needs to be validated in the future; secondly, another major drawback is the current lack of satisfactory human NASH data reports, so the core genes applicable to human NASH are difficult to compare. In addition, the complexity and diversity of human NASH compared to mouse models requires further study to ensure the reliability of conclusions across species. In addition, the microenvironment and immune response of the human liver may be very different, which may affect the expression and role of core genes. In the future direction of using machine learning to study NASH, the acquisition of high-quality human NASH single-cell RNA sequencing data should be improved and rigorous experiments should be conducted to validate the core genes identified in this study. This will facilitate the understanding and treatment of this complex disease and further improve the effectiveness of the application of machine learning methods.

Overall, our study provides new insights into the pathogenesis of NASH and demonstrates the strong potential of deep learning and scRNA-seq in disease research. These techniques not only enhance our understanding of disease processes but also pave the way for the development of novel diagnostic and therapeutic strategies in the future.

## 5 Conclusion

In summary, this study elucidated the mouse model of NASH at the single-cell level, potentially enhancing our comprehensive understanding of this disease. We discovered key features in the NASH liver, including a dramatically reduced proportion of liver sinusoidal endothelial cells and a significant trend where monocytes developed into Kupffer cells, underscoring the critical role of macrophages in NASH progression. Importantly, core genes, such as *Anax1* and *Gpnmb* were identified in association with NASH development and immune activation. These findings provided new insights into the molecular mechanisms underlying NASH and may pave the way for exploring potential therapeutic targets, making a significant contribution to the treatment of NASH.

## Ethics approval and consent to participate

Not applicable.

## Consent for publication

Not applicable.

## Availability of data and materials

All data generated or analyzed during this study are downloaded from GEO database.

## Funding

This research was funded partly by the 10.13039/501100001809National Natural Science Foundation of China (82070638, 82270697 and 82370517), the 10.13039/501100012245Science and Technology Planning Project of Guangdong Province of China (2021B1212040016), GuangDong Basic and Applied Basic Research Foundation (2023A1515012574); Jiangsu Provincial Medical Key Discipline Cultivation Unit (JSDW202229), and the Grant for International Joint Research Project of the Institute of Medical Science, the 10.13039/501100004721University of Tokyo.

## CRediT authorship contribution statement

**Yu-Mu Song:** Writing – review & editing, Writing – original draft. **Jian-Yun Ge:** Writing – review & editing, Funding acquisition, Conceptualization. **Min Ding:** Data curation, Formal analysis, Investigation, Methodology, Validation, Writing – review & editing. **Yun-Wen Zheng:** Writing – review & editing, Validation, Supervision, Resources, Project administration, Funding acquisition, Conceptualization.

## Declaration of competing interest

The authors declare the following financial interests/personal relationships which may be considered as potential competing interests:Yun-Wen Zheng reports article publishing charges, equipment, drugs, or supplies, and travel were provided by National Natural Science Foundation of China. Yun-Wen Zheng reports article publishing charges, equipment, drugs, or supplies, and travel were provided by Jiangsu Provincial Medical Key Discipline Cultivation Unit. Yun-Wen Zheng reports article publishing charges, equipment, drugs, or supplies, and travel were provided by the Grant for International Joint Research Project of the Institute of Medical Science, the 10.13039/501100004721University of Tokyo. If there are other authors, they declare that they have no known competing financial interests or personal relationships that could have appeared to influence the work reported in this paper.
