# Supplementary figures and images for: Key factor screening in mouse NASH model using single-cell sequencing combined with machine learning
Source: Heliyon. 2024 Jun 25;10(13):e33597. doi: 10.1016/j.heliyon.2024.e33597 (PMC11260934; doi:10.1016/j.heliyon.2024.e33597)

A

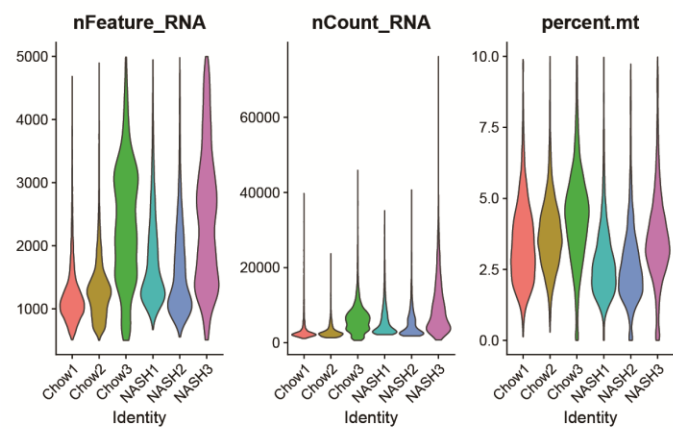

B

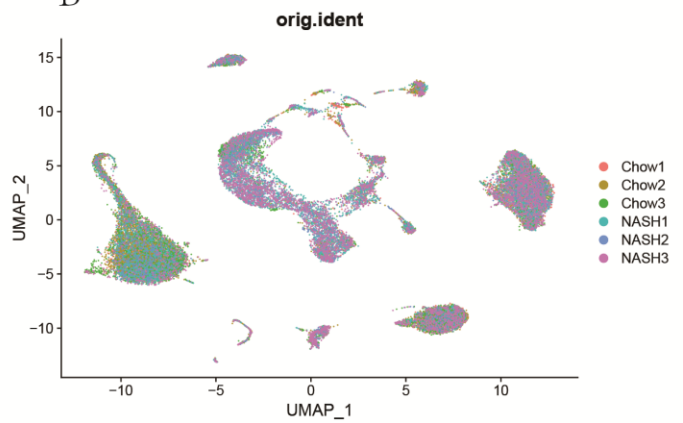

C

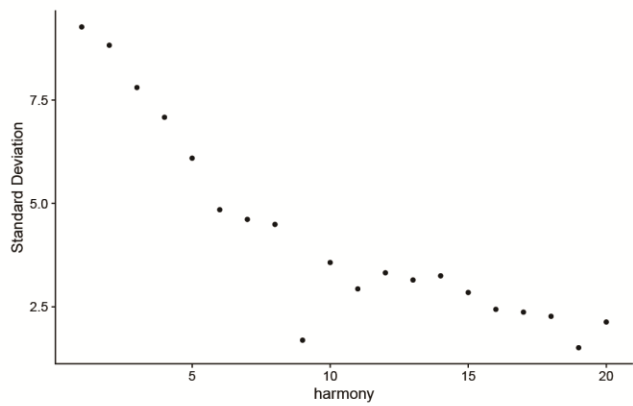

D

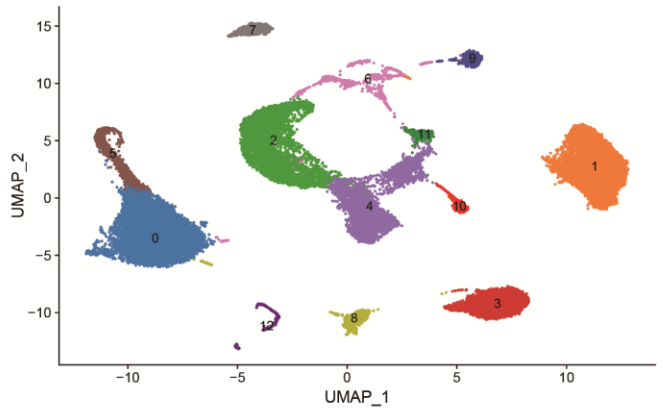

Supplementary Figure S1

Supplement: Multimedia component 5 [file mmc5.pdf]
